# Medical Department University of Texas

**Published:** 1894-05

**Authors:** 


### EDITORIAL DEPARTMENT.

F. E. DANIEL, M. D., Editor.
S. E. HUDSON, M. D., Managing Editor.
A. J. SMITH. M. D., Galveston, Associate Editor.

EDITORIAL STAFF:

PROF. J. E. THOMPSON, M. D., Texas Medical College, Galveston; Surgery.
PROF. WM. KEILLER, M. D., Texas Medical College, Galveston; Obstetrics and Gynecology.
PROF. DAVID CERNA, M. D., Texas Medical College, Galveston; Therapeutics.
PROF. A. J. SMITH, M. D., Texas Medical College, Galveston; Medicine.
DR. ISADORE DYER, Tulane University; Dermatology.
DR. R. H. L. BIBB, Saltillo, Mexico; Foreign Correspondent.
DR. ROBT. MORRIS, Charity Hospital, N. O.; Clinical Reports

### MEDICAL DEPARTMENT UNIVERSITY OF TEXAS.

On the evening of May 1, 1894, in Harmony Hall, at Galveston, the third annual graduation exercises of the Medical Department of the State University were held. After prayer by the Rev. Dr. Scott, Dr. Allen J. Smith delivered the annual address to the graduating class, upon the influence of medical studies upon religious thought. He acknowledged the existence of a basis for the prevalent idea that medical men are of skeptical tendency in religious matters; but he strongly opposed the assertion that medical studies are conducive to absolute atheism, which he regarded as really largely due to personal mental peculiarities on the part of the atheist. He did not hesitate to state that a large proportion of the profession is unorthodox in the details of religion, but asserted that for the most part this group offers a brilliant and intelligent faith in Deity, born in doubt, but full of the truest elements of worship. The unreasoning, taught to regard man as of divine origin and gifted with elements of the divine nature, might, when the realization of the animal nature of man is thrust upon them, be, by revulsion, hurried to the negations of atheism; but normal, even minds, which constitute the bulk of the profession, are but led to recognize in this very idea the evidences of a higher power. Regarding man as an animal with no essential differences of structure from the higher grades of his inferiors, we may be permitted the same liberty of discussion of his higher functions as we would employ in the demonstration of ordinary animal functions. As far as can be determined, the physiologic impossibility of a nerve cell without communication with other cells, or with the periphery, may be maintained without exception. The motor and sensory nature of certain nerve cells is established by experimental demonstration; the ideational function of nerve cells is held on weaker basis as proof, but can scarcely be doubted when cerebral lesions and mental faults are found associated. Believing, then, that thought cells are nerve cells just as other nerve cells, believing that isolated nerve cells without peripheral or inter-communication are functional impossibilities, we are bound to accept the view that thought cells, like motor and sensory nerve cells, are in communication with each other and with the periphery—that they receive and register impressions, and from these formulate ideas and originate impulses. The gradual growth of thought from infancy onward, speaks strongly of such a nature. The person who has never received impressions in relation to pain can never appreciate or think of pain; the individual without impressions of color is unable to think of color. The memory is impossible without impressions to be remembered; imagination is but the rearrangement of impressions in new relations. Reason is but argumentation from established facts—from established impressions; the establishment of fact does not rest with man, only its discovery—thus, two and two made four before man discovered the fact. Even judgment, the small voice of conscience, the realization of right and wrong, depends upon surroundings; the Hindoo mother who hurls her infant into the Ganges, has an approval of conscience, as well as the doer of the most Christian act. If man in his higher nature, in the part which really approaches or is the soul, is thus constituted that without impressions he may grow to the age of Methuselah and have no thought or active nervous function, he is not an automaton, he is not a free agent, but one dominated, guided and controlled by an omnipresent, an omniscient, an all-powerful agent external to him—by one above and beyond his comprehension, inscrutible and infinite—by a God. This removal of responsibility from man and its reference to Divinity, is not in the least inexplicable or unjustifiable when one realizes the grand purpose of the greatest good to the greatest number. The thousands whose deaths in the Inquisition horrify the world after centuries have passed, have put paid a slender price for the happiness of religious freedom for millions and millions now. The awful records of the plagues only stand as the warnings, the commands to appreciate and practice the blessings of preventive medicine. The groans and the pains, the horrors of battle and of blood, the misfortunes of life do not count for the individual where blessings may be thereby brought to the multitudes. He who gives and takes knows the happiness and the misery of each, and death may be a boon when earth's experiences are run; and so with reason and with grace the responsibility may be left with God.

So too, remembering that the law of the universe demands the greatest good for the greatest number, and that the irresponsible individual in his crime meets as irresponsible masses of his fellows whom he wrongs, when the claim of Divine responsibility for crime is advanced, the equally Divine right, responsibility and duty of public protection is to be remembered, and the laws of society vindicated.

At the termination of this address, Hon. T. D. Wooten, M. D., President of the Board of Regents of the University of Texas, conferred, at the request of the Dean of the Medical Faculty, the degree of Doctor of Medicine upon Malone Duggan, Walter N. John, George Sparks, Jacob H. Sampson, and Ernest A. Thompson.

Mr. Richard A. Goeth, who had complied with all the class requirements for graduation, but who lacked nearly a year of the legal majority, was given a certificate of his eligibility for the degree at the next commencement.

The State Medical Association medal for the best final examination, the Faculty medal for the highest general average in all the branches of the entire course, and the J. M. Kellar medal for the best examination in obstetrics and gynecology, were all awarded Dr. J. H. Sampson. Mr. J. T. Moore, of the first year class, was awarded the prize in histology offered annually by the professor of pathology, the prize this year being a copy of the last edition of Carpenter on the Microscope and its Revelations. A large list of distinctions were announced in the different classes, after which the exercises were closed with benediction by Rev. Dr. Scott.

The session of the school just closed has been highly successful, not only in the very large increase in the number of the students, but as well in the earnestness of work which has marked the entire curriculum. The Regents, in their session in Galveston on April 30th and May 1st, announced or confirmed several important changes in the methods of the school. Hereafter graduates from reputable medical schools, if residents of the

State of Texas, will be admitted to post-graduate study without payment of fees, unless laboratory courses are undertaken, or the degree of the institution applied for. In the former case, the usual fee of five dollars for each laboratory attended, in the latter, a fee of ten dollars will be charged.

Dr. Seth M. Morris, Professor of Chemistry, whose tenure of office has heretofore been from year to year, was elected to his chair upon the same basis as his colleagues, without any defined limit of tenure.

A Demonstratorship of Normal Histology was created, to be attached to the chair of pathology, and Dr. William Gammon, a graduate of the school last year, was appointed to the position.

It is to be noticed, too, that the coming session has been lengthened to May 15, 1895, in order to provide a full seven months course of instruction and at the same time have sufficient time for proper examination at the end of the term. In the same line of advance it is to be announced that students will not be permitted to matriculate later than October 15, 1894, the marked difficulty of catching up with the class for those students matriculating at a later date making this step an imperative one.

